# Gender as a moderator of the relationships among body composition, physical activity, basal metabolic rate, and BMI in Taiwanese university students

**DOI:** 10.1186/s12889-026-26810-w

**Published:** 2026-03-07

**Authors:** Hsiang-Ping Wu, Hsiang-Yun Lan, Yu-Lun Tsai, Yu-Ting Chen, Huai-Shuo Huang, Jen-Jiuan Liaw, Yue-Cune Chang

**Affiliations:** 1 Department of Nursing, Chung-Jen Junior College of Nursing, Health Sciences and Management, Chiayi County, Taiwan; 2 College of Nursing, National Defense Medical University, Taipei City, Taiwan; 3 Department of Nursing, Tri-Service General Hospital, Taipei City, Taiwan; 4 Department of Nursing, College of Healthcare and Management, Asia Eastern University of Science and Technology, New Taipei City, Taiwan; 5 Graduate Institute of Nursing, National Defense Medical University, Taipei City, Taiwan; 6 Department of Mathematics, Tamkang University, Taipei City, Taiwan

**Keywords:** Gender, Body composition, Physical activity, Basal metabolic rate, Overweight, obesity

## Abstract

**Background:**

Early adulthood exhibits rapid weight gain. While body composition, physical activity (PA), and basal metabolic rate (BMR) drive weight regulation, gender complicates these interactions. It remains unclear if gender moderates the associations of these factors with BMI. Clarifying this aids obesity assessment and supports gender-specific health management.

**Aim:**

This study examined the associations between body composition, PA, and BMR with BMI, and explored whether gender moderates these relationships in Taiwanese university students.

**Method:**

This cross-sectional study recruited 250 Taiwanese university students (125 males and 125 females, aged 18–24) from various faculties in northern Taiwan using convenience sampling. Body composition, including body fat percentage (BFP), bone mass (BM), muscle mass (MM), and visceral fat level (VFL), along with BMR were measured using dual-frequency bioelectrical impedance analysis (BIA) under standardized conditions. PA was assessed using the International Physical Activity Questionnaire-Short Form (IPAQ-SF). Multiple linear regression analyses with interaction terms were utilized to examine associations and the moderating role of gender.

**Results:**

BFP, BM, MM, VFL, and BMR were significantly correlated with BMI. In the regression model, VFL (β = 0.535, *p* < 0.001), BFP (β = 0.436, *p* < 0.001), BMR (β = 0.309, *p* < 0.001), and gender (B = -0.033, *p* < 0.050) showed significant associations with BMI. Notably, gender significantly moderated the relationships between VFL and BMI (B = 0.014, *p* < 0.001), and between BMR and BMI (B = 2.29E-04, *p* = 0.005).

**Conclusion:**

Gender, body fat, and BMR were significantly associated with BMI in young adults. Notably, gender moderated the associations of VFL and BMR with BMI, with stronger effects observed in women than in men. These findings indicate that clinicians should consider gender differences when examining the associations between VFL and BMI as well as between BMR and BMI. Consequently, incorporating BFP, VFL, and BMR alongside traditional measures (BW and BMI) is essential to optimize obesity assessment. Furthermore, this study highlights the need for gender-specific strategies to enhance obesity and metabolic health management in university students.

**Supplementary Information:**

The online version contains supplementary material available at 10.1186/s12889-026-26810-w.

## Introduction

The global burden of excess weight and obesity remains a critical public health challenge [1, 2]. In 2022, an estimated 2.5 billion adults (43%) were classified as overweight and 890 million (16%) as obese, with comparable global prevalence between men (43%) and women (44%) [3]. Notably, early adulthood is recognized as a crucial turning point for weight trajectory and subsequent health risk [4]. This university stage exhibits faster rates of weight gain [5] and faces the highest risk of transitioning to overweight and obesity over a 10-year period [2]. In Taiwan, marked gender disparities have been observed among individuals aged 18–24 years, with a substantially higher prevalence of overweight and obesity among men (48.9%) than women (26.4%) [6]. Despite these gender differences in obesity prevalence during early adulthood, it remains unclear whether gender moderates the associations between obesity-related physiological and behavioral factors and body mass index (BMI) in this high-risk age group.

BMI is a widely adopted anthropometric measure for assessing overweight and obesity, favored for its cost-effectiveness, accessibility, and ease of calculation [1, 7, 8]. However, BMI has a fundamental limitation: it cannot distinguish between the contributions of fat mass (FM) and fat-free mass (FFM) to total body weight (BW) [9, 10]. This can result in misclassification, particularly in young males with high muscle gain, where elevated BMI does not necessarily indicate excess adiposity [10]. To enhance diagnostic accuracy, the literature suggests that BMI should be integrated with body fat and other measures of body composition [10]. Consequently, a clear understanding of the association between BMI and body composition in male and female university students is essential for accurate clinical identification of obesity and advancing research [9].

Body composition is defined by the proportions of its various components [e.g., FM, bone mass (BM), muscle mass (MM), visceral fat level (VFL), and fluids [10–13]. Body composition is strongly influenced by biological sex, especially after puberty, when hormonal regulation leads to pronounced sexual dimorphism [14, 15]. Women generally have higher overall body fat percentage (BFP) and greater subcutaneous fat, whereas men have higher BW—including BM and MM, and greater visceral fat deposition. This elevated VFL represents a key risk factor for obesity progression [12, 14, 15]. BM reaches its peak in university students aged 18–23 years and is positively correlated with BW [16–19]. Furthermore, increases in MM are the primary contributors to BMI gains in male students, whereas female students experience a more rapid increase in BFP [10]. The association between body composition and BMI highlights the need to integrate both measures when assessing obesity in university students [9, 10].

Physical Activity (PA) is a crucial determinant in preventing and managing obesity by increasing energy expenditure and facilitating the restoration of energy balance [20]. PA is defined as any skeletal muscle-produced movement that expends energy [21]. Large-scale empirical evidence demonstrates an inverse association between PA and BMI in populations aged 40–69 years [22]. However, study findings remain inconsistent. Some studies report that high-intensity PA tends to reduce adiposity and attenuate weight gain [21, 23]. The other cross-sectional study demonstrates that exercise may not effectively aids weight control in individuals with overweight or obesity [24]. This complex associations are further compounded by gender differences in intensity of PA [25].

Basal metabolic rate (BMR) is the primary energy expenditure source [26, 27]. Due to increased BW accompanied by greater skeletal MM, BMR tends to increase [28]. Consequently, young adults with obesity typically exhibit higher BMR than lean adults [28]. Nevertheless, the role of BMR in weight regulation still remains debated. Some studies suggest that the lower BMR only has a small significant impact on the rate of weight gain [29, 30], while others report that no significant difference in weight gain between the adults with low BMR and those with high BMR [31]. Critically, even after adjusting for body composition, men exhibit higher BMR due to more MM than women [11, 27]. Therefore, gender is a major predictor of BMR [11, 26].

The inconsistency in the above study findings drove us to further explore the associations among body composition, PA, BMR, and BMI. In addition, these findings indicate that BMI is shaped by complex interactions among body composition, PA, BMR, and gender. However, few studies have explored gender roles in the relationships among body composition, PA, BMR, and BMI. Therefore, this study aimed to examine the associations of body composition, PA, and BMR with BMI, and to explore whether gender moderates these relationships in Taiwanese university students. Based on the literature evidence, we hypothesized that: (1) body composition components (FM, MM, BM, and VFL), PA, and BMR are significantly associated with BMI in Taiwanese university students; and (2) gender can moderate the associations between these factors of body composition, PA, and BMR with BMI.

## Materials and methods

### Design

This study used cross-sectional design to explore the moderate roles of gender on the relationships among body composition, PA, and BMR and BMI [32]. The measurements include body composition parameters (FM, BM, MM, and VFL), PA, BMR, and BMI among university students in Taiwan. Demographic information, specifically age and gender, were collected because these variables are known to be associated with variation in body composition parameters, PA, BMR, and BMI.

### Sample and setting

The study sample comprised university students from various faculties at a university in northern Taiwan. This population was selected because these students are transitioning from adolescence to young adulthood, a period marked by substantial biological, physiological, and hormonal changes that differ by sex [14, 15]. Early adulthood represents a pivotal developmental stage characterized by increasing autonomy over lifestyle choices, making it a critical window for examining the determinants of obesity and weight regulation. Health behaviors and lifestyle patterns established during this stage may have profound and lasting effects on subsequent health outcomes [5]. According to health promotion statistics in Taiwan, individuals aged 18–24 years constitute a distinct population category, which informed the present study’s focus on early adulthood [6]. Given the heightened risk of body weight gain and obesity during this life stage, preventive efforts among university students are imperative. Therefore, this study specifically targets individuals in early adulthood.

Participants were recruited using a convenience sampling approach [32]. Recruitment was personally conducted by the first author through campus-wide announcements, classroom-based invitations across multiple faculties, and bulletin board postings. All faculties were eligible for participation, and no restrictions were placed on academic discipline, in order to reduce discipline-specific bias. The following criteria included: (a) students aged 18–24 years; (b) enrolled for at least six months; (c) no chronic diseases or functional impairments; (d) no mental health issues; and (e) provision of informed consent. Exclusion criteria were: (a) presence of overt endocrine or metabolic diseases diagnosed by the certified physicians (e.g., hypertension, diabetes, hypothyroidism); or (b) regular use of medications known to influence energy metabolism.

### Participant recruitment and enrollment

**Fig. 1:**
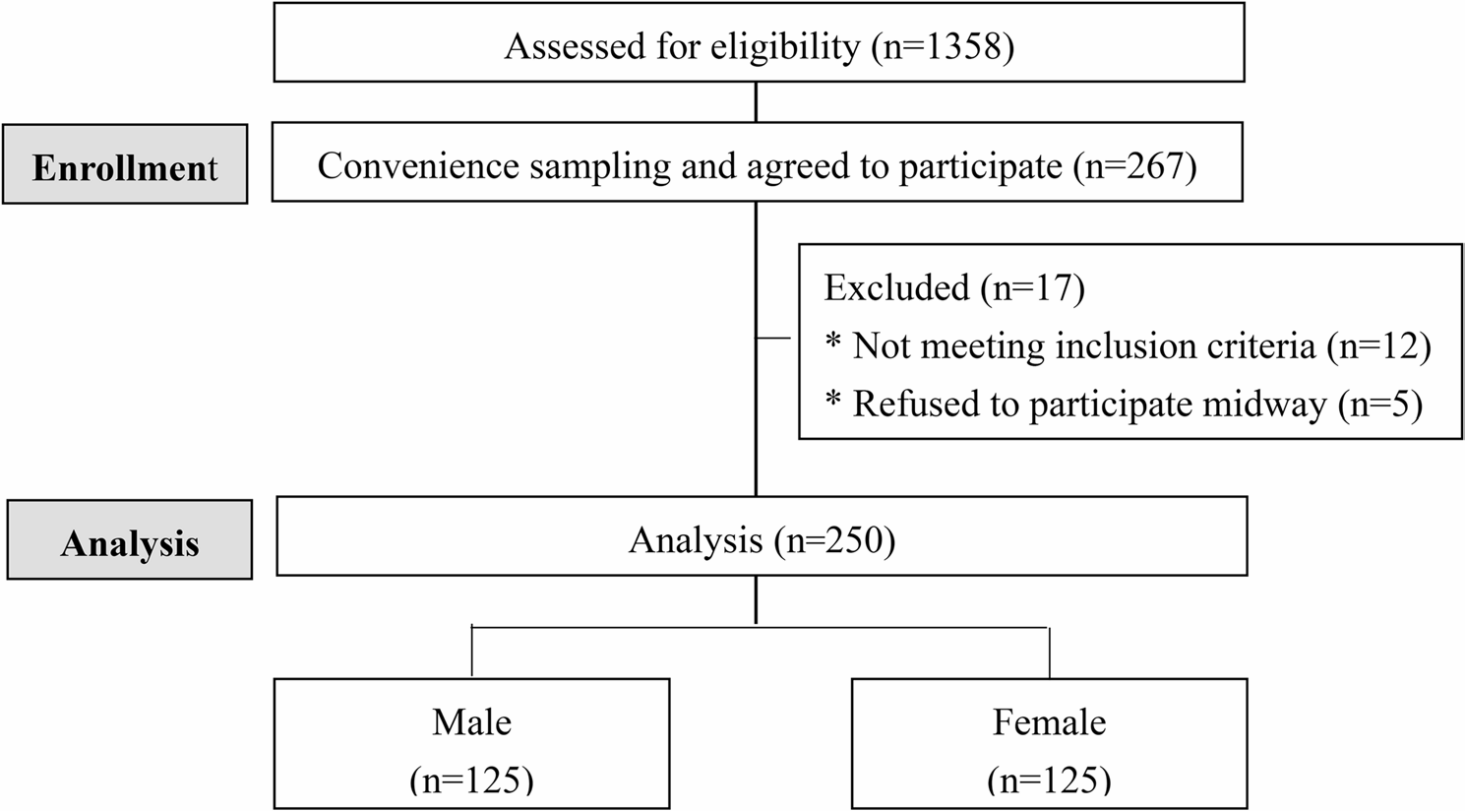
Flowchart of participant recruitment

The “Linear multiple regression: Fixed model, R² deviation from zero” in G*Power 3.1.9.2 was used to estimate the sample size needed for this study. By setting effect size = 0.07, α = 0.05, power = 0.80, number of predictors = 11, the estimated sample needed in this study was 251 [33]. During the six-month recruitment period (July–December 2020), 267 students provided informed consent and underwent eligibility screening based on self-reported health status. Seventeen participants were subsequently removed: 12 for not meeting eligibility criteria, and 5 withdrew consent. Our final sample of 250 participants (125 men, 125 women) entered the analyses and achieved the threshold, ensuring robust statistical power. The enrollment process is summarized in Fig. 1.

### Measurement of body composition, BMR and BMI

Body composition and BMR were assessed using the Segmental Body Composition Monitor (Model BC-545 N, TANITA Corporation, Tokyo, Japan), following the manufacturer’s guidelines [34]. This device integrates Bio-electrical Impedance Analysis (BIA) with Advanced Dual Frequency Inner Scan technology. To ensure measurement accuracy, participants followed a strict pre-assessment protocol. Assessments were conducted in the early morning (between 07:00 and 08:30), required an overnight fast (after 12:00 AM) before data collection, and participants abstained from strenuous exercise, caffeine, and alcohol for at least 24 h before the assessment [11, 35]. In addition, they voided their bladder and bowels prior to measurement and wore only lightweight clothing [36].

Body height was first measured to the nearest 0.01 m using a wall-mounted stadiometer. For the BIA assessment, participants stood barefoot with heels aligned on the platform electrodes and grasped the handheld electrodes, maintaining a stable, upright posture until the reading was complete [11, 36]. Two consecutive measurements were taken and averaged for analysis. Two trained researchers followed the instrument manual to record the following metrics: (1) BW, measured to the nearest 0.1 kg; (2) BFP, FM relative to total BW (standard ranges of 10–21% for young men and 20–34% for young women); (3) BM, measured in kilograms (typically 2.5–3.2 kg in young men and 1.8–2.5 kg in young women); (4) MM, total MM including intramuscular water (typically 50–70 kg in young men and 30–50 kg in young women); (5) VFL, assessed on a 1–59 scale (10–14 elevated; ≥15 excessive); and (6) BMR, minimum resting energy expenditure (~ 1550 kcal/day in men; ~1210 kcal/day in women) [34]. BMI was simultaneously calculated as the participant’s weight (in kilograms) divided by the square of their height (in meters) (kg/m^2^). Based on the Health Promotion Administration of Taiwan’s guidelines, overweight was defined as BMI = 24.0–26.9 kg/m^2^, and obesity was defined as BMI ≥ 27.0 kg/m^2^ [37].

### Measurement of PA

PA was measured using the International Physical Activity Questionnaire-Short Form (IPAQ-SF) [38], a 7-item, self-administered tool that captures time spent on walking, moderate, and vigorous activities across four domains (leisure, domestic, work, and transport) during the preceding seven days. The IPAQ-SF was translated into Taiwan-version by Liou et al. in 2008 and the validity and reliability were examined [38]. Total PA was derived by summing weighted activity durations, expressed in MET-min/week. The IPAQ-SF has been validated in Taiwan and demonstrates strong psychometric properties, including high content validity (CVI = 0.994), excellent language equivalence between the Chinese and English versions (0.992), and acceptable test–retest reliability, as indicated by an intra-class correlation coefficient (ICC = 0.704) [38].

### Procedures

The study was approved by the Institutional Review Board (IRB) of Tri-Services General Hospital (IRB No. [B202005080]). Prior to data collection, the principal investigator trained all research personnel and prepared the Segmental Body Composition Monitor and the IPAQ-SF. To mitigate the potential for a vertical power dynamic between researchers (teachers) and participants (students), recruitment was conducted by trained assistants following standard procedures to protect participant autonomy. The first author provided a detailed explanation of the study and potential risks, and written informed consent was obtained from each eligible young adult before any data collection began.

The trained research assistants conducted all recruitment following standardized procedures. The first author initially approached eligible young adults to provide a detailed explanation of the study, its procedures, and potential risks. Written informed consent was secured from all participants, and data collection commenced only after the signed consent form was obtained.

### Data analysis

Statistical analyses were performed using IBM SPSS Statistics (version 26.0). Descriptive statistics are presented as mean ± standard deviation (SD) for continuous variables and as number (percentage) for categorical variables. Gender differences were examined using independent-samples t tests for continuous variables and chi-square tests for categorical variables. Bivariate associations among body composition parameters, PA, BMR, and BMI were evaluated using Pearson’s correlation coefficients. Prior to inferential analyses, normality of continuous variables was evaluated using visual inspection of histograms and normal Q–Q plots. BMI distribution appeared slightly skewed to the right; therefore, the natural logarithm of BMI (ln[BMI]) was applied to improve normality and stabilize variance.

Multiple linear regression analyses were conducted to examine the independent and moderating effects of gender, body composition components, PA, and BMR on ln[BMI]. All models were adjusted for university faculty. Multicollinearity among predictors was assessed using variance inflation factors (VIFs) and tolerance values. An initial full model (Model 1) included all body composition predictors, PA, and BMR. Model 1 exhibited severe multicollinearity, particularly for MM (VIF = 38.191) and BMR (VIF = 27.512). To address these issues, Model 2 was derived using stepwise selection to identify the strongest predictors of ln[BMI]. In Model 2, VIF values remained within acceptable limits (VIF < 5.00), as variables failing to significantly improve model fit were systematically removed to ensure a robust and parsimonious final model. Despite the known limitations of stepwise selection, results from both the full and parsimonious models were reported for transparency. In addition, multiple regression analyses were performed using mean-centered continuous predictors to evaluate the independent contributions of gender, body composition, PA, and BMR to InBMI. Model fit was evaluated using R², adjusted R², F-statistics, and residual diagnostics. Multiple interaction terms were tested as part of a hypothesis-driven but exploratory moderation analysis; therefore, no formal correction for multiple comparisons was applied. Statistical significance was determined using two-tailed tests with a threshold of *p* < 0.05.

## Results

### Characteristics of young adults

Table 1 compares characteristics among the 250 participants by sex. Continuous variables were analyzed using independent-samples t-tests. The results suggest mean age did not differ significantly between men and women (20.53 ± 1.43 years; *p* > 0.05). Men exhibited significantly higher mean values than women across nearly all physical measurements (*p* < 0.05 for all): (1) Anthropometrics: Men were significantly taller (172.91 ± 8.04 vs. 161.59 ± 5.33 cm), heavier (68.94 ± 9.72 vs. 55.26 ± 6.91 kg), and had a higher BMI (22.85 ± 2.73 vs. 21.23 ± 2.30 kg/m²). (2) Body composition: Men had greater BM (2.95 ± 0.33 vs. 2.27 ± 0.27 kg), MM (53.44 ± 6.09 vs. 37.54 ± 3.65 kg), and VFL (5.55 ± 3.13 vs. 3.31 ± 1.69 level). Conversely, BFP was significantly lower in men (17.60 ± 5.11%) than in women (28.08 ± 5.18%). (3) Metabolic measures: Men reported significantly higher PA (4138.94 ± 3808.51 vs. 2577.04 ± 2083.15 MET-min/week) and BMR (1651.61 ± 206.79 vs. 1233.47 ± 104.67 kcal).

**Table 1:** The demographic characteristics of the participants by gender

**Variable**	**Overall (n = 250)** **mean ± SD**	**Male (n = 125)** **mean ± SD**	**Female(n = 125)** **mean ± SD**	t /c^2^	***p*** **-value**
Age (years)	20.53 ± 1.43	20.37 ± 1.43	20.70 ± 1.44	-1.823	0.069^a^
Body height (cm)	167.23 ± 8.85	172.91 ± 8.04	161.59 ± 5.33	13.110	< .001^a^
Body weight (kg)	62.10 ± 10.86	68.94 ± 9.72	55.26 ± 6.91	12.826	< .001^a^
Body composition					
BFP (%)	22.82 ± 7.34	17.60 ± 5.11	28.08 ± 5.18	-16.08	< .001^a^
BM (kg)	2.61 ± 0.45	2.95 ± 0.33	2.27 ± 0.27	17.94	< .001^a^
MM (kg)	45.49 ± 9.41	53.44 ± 6.09	37.54 ± 3.65	25.04	< .001^a^
VFL (level)	4.43 ± 2.75	5.55 ± 3.13	3.31 ± 1.69	7.02	< .001^a^
PA (MET-min/week)	3346.33 ± 3148.27	4138.94±3808.51	2577.04±2083.15	3.59	< .001 ^a^
BMR (kcal)	1443.38 ± 265.90	1651.61 ± 206.79	1233.47 ± 104.67	20.15	< .001^a^
BMI (kg/m^2^)	22.04 ± 2.65	22.85 ± 2.73	21.23 ± 2.30	5.07	< .001^a^
BMI＜18.5	17 (6.8)	5(4.0)	12 (9.6)	16.39	=.001 ^b^
18.5 ≤ BMI＜24	169 (67.6)	75(60.0)	94 (75.2)		
24≤ BMI＜27	45 (18.0)	30(24.0)	15 (12.0)		
BMI≧27	19 (7.6)	15(12.0)	4 (3.2)		
Faculties				42.55	< .001^b^
medicine	145 (58.0)	91 (72.8)	54 (43.2)		
dentistry	18 (7.2)	7 (5.6)	11 (8.8)		
pharmacy	13 (5.2)	9 (7.2)	4 (3.2)		
nursing	66 (26.4)	12 (9.6)	54 (43.2)		
public health	7 (2.8)	6 (4.8)	1 (0.8)		
missing	1 (0.4)	0	1 (0.8)		

Note: *SD* standard deviation, *BMI* body mass index, *BFP* body fat percentage, *BM* bone mass, *MM* muscle mass, *VFL* visceral fat level, *PA* physical activity, *BMR* basal metabolic rate. *p*-value a: by t test; *p*-valueb: by Chi-Square test

The categorical variables were compared using chi-square tests. The results suggest the distribution of BMI categories differed significantly by sex (*p* = 0.001); men were distributed as underweight (4.0%), healthy weight (60.0%), overweight (24.0%), and obese (12.0%), whereas women were more frequently classified as underweight (9.6%) or healthy weight (75.2%), with lower proportions of overweight (12.0%) and obesity (3.2%). The distribution of BMI categories differed significantly between men and women (*p* = 0.001). Faculty also differed significantly by sex (*p* < 0.001), with a larger proportion of male participants majoring in medicine (72.8%), while female participants were primarily distributed between medicine (43.2%) and nursing (43.2%).

### Correlation analysis of each variable

Pearson’s correlation analysis (Table 2) was employed to assess the bivariate relationships between BMI and various body composition metrics, PA, and BMR within the university students. Furthermore, BMI was significantly and positively correlated with all other physiological variables (all *p* ≤ 0.004): The strongest correlation was observed with VF (*r* = 0.869). Other strong positive correlations included BMR (*r* = 0.621), BM (*r* = 0.546), and MM (*r* = 0.526). Weak positive correlations were found with BFP (*r* = 0.307) and PA (*r* = 0.200).

**Table 2:** Pearson‘s correlation between gender, body composition, PA, BMR and BMI

Variable	BMI					
	Overall		Male		Female	
	*r*	p-value	*r*	p-value	*r*	p-value
Body composition						
BFP	0.307	< .001	0.784	< .001	0.798	< .001
BM	0.546	< .001	0.496	< .001	0.513	< .001
MM	0.526	< .001	0.629	< .001	0.341	< .001
VFL	0.869	< .001	0.912	< .001	0.790	< .001
PA	0.200	0.004	0.145	0.151	0.115	0.248
BMR	0.621	< .001	0.685	< .001	0.616	< .001

Note: *BMI *body mass index, *BFP *body fat percentage, *BM *bone mass, *MM *muscle mass, *VFL *visceral fat level, *PA *physical activity, *BMR *basal metabolic rate, r correlation coefficient

Gender coded: 1 for males, 2 for females

*p < 0.05, **p < 0.01, ***p < 0.001

When the data were stratified by gender, the correlation patterns were more specific: In both men and women, BMI retained strong, positive correlations with BFP, BM, MM, VFL, and BMR (all *p* < 0.001). The strongest correlations with BMI were observed for VFL (men: *r* = 0.912; women: *r* = 0.790) and BFP (men: *r* = 0.784; women: *r* = 0.798). Crucially, PA was not significantly correlated with BMI in either men (*r* = 0.145, *p* > 0.05) or women (*r* = 0.115, *p* > 0.05). This suggests that the weak positive relationship observed in the combined sample was confounded by the inclusion of both gender.

### Associations among body composition, PA, and BMR on BMI

Multiple linear regression analysis was conducted to determine the independent contributions of gender, body composition components, PA, and BMR to BMI. The results using original metrics are presented in Table 3, while the results from the analysis using mean-centered variables are provided in Supplementary Table S1. The natural logarithm of BMI (ln[BMI]) served as the dependent variable in all models, with all analyses adjusted for participants’ university faculty.

**Table 3:** Multiple regression analysis to test for the independent contributions of gender, body composition, PA, BMR to InBMI, after adjusting for faculties

Variable	*B*	SE	β	t	*P*	Collinearity		Model fit indices			
						Tolerance	VIF	*R*²	*adjusted R²*	F	*P*
Model 1								0.852	0.845	135.711	< 0.001
Gender	-0.030	0.018	-0.125	-1.714	0.088	0.147	6.807				
Body composition											
BFP (%)	0.008	0.001	0.453	6.622	< 0.001	0.168	5.961				
BM (kg)	0.031	0.018	0.118	1.660	0.099	0.156	6.398				
MM (kg)	0.001	0.002	0.083	0.480	0.632	0.026	38.191				
VFL (level)	0.023	0.003	0.530	8.031	< 0.001	0.180	5.544				
PA (MET-min/week)	1.68E-06	1.14E-06	0.044	1.478	0.141	0.896	1.116				
BMR (kcal)	5.55E-05	6.46E-05	0.126	0.859	0.391	0.036	27.512				
Model 2								0.847	0.844	267.837	< 0.001
Gender	-0.033	0.017	-0.135	-1.976	0.050	0.169	5.904				
Body composition											
BFP (%)	0.007	0.001	0.436	7.049	< 0.001	0.207	4.832				
VFL (level)	0.023	0.003	0.535	8.720	< 0.001	0.210	4.763				
BMR (kcal)	1.36E-04	2.71E-05	0.309	5.005	< 0.001	0.208	4.818				

Note: Dependent variable: natural logarithm of BMI (InBMI); Predictors: *BFP *body fat percentage, *BM *bone mass, *MM *muscle mass, *VFL *visceral fat level, *PA *physical activity, *BMR *basal metabolic rate, *SE *standard error, *95% CI* 95% confidence interval

*p < 0.05, **p < 0.01, ***p < 0.001

#### Model 1: initial assessment

Model 1 included gender, all body composition components, PA, and BMR. In this model, only BFP (β = 0.453, *p* < 0.001) and VFL (β = 0.530, *p* < 0.001) emerged as statistically significant independent predictors of ln(BMI). The remaining variables, gender (β = −0.125, *p* = 0.088), BM (β = 0.118, *p* = 0.099), MM (β = 0.083, *p* = 0.632), PA (β = 0.044, *p* = 0.141), and BMR (β = 0.126, *p* = 0.391), did not reach statistical significance. Regarding model fit indices for Model 1, the regression model accounted for 85.2% of the variance in lnBMI (R² = 0.852; adjusted R² = 0.845), and the overall model was statistically significant (F = 135.711, *p* < 0.001).

#### Model 2: final predictive model for BMI (stepwise selection)

Model 2 was established using a stepwise selection procedure. This approach retained only variables that made a significant contribution to predicting ln(BMI) within the young adult cohort. After adjusted for the effects of faculties in the model, the BMI (in log scale) of female is, on average, 0.033 unites borderline significantly lower than that of male (B = − 0.033, *p* = 0.050). VFL emerged as the strongest positive predictor (β = 0.535; *p* < 0.001), followed by BFP (β = 0.436; *p* < 0.001) and BMR (β = 0.309; *p* < 0.001). Notably, BM (*p* = 0.083) MM (*p* = 0.478), PA (*p* = 0.139) were excluded from the final model as they failed to meet the retention criteria. The regression model 2 was statistically significant (F = 267.837, *p* < 0.001) and accounted for 84.7% of the variance in lnBMI (R² = 0.847; adjusted R² = 0.844).

### Direct effects on ln(BMI)

A multiple linear regression analysis was conducted to examine the moderating effect of gender on the relationships among body composition, PA, BMR, and BMI (Table 4). The analysis used the natural logarithm of BMI and was adjusted for participants’ university faculties. Before testing moderation, the main effects showed that BFP (B = 0.019, *p* < 0.001), BM (B = 0.190, *p* < 0.001), MM (B = 0.012, *p* < 0.001), VFL (B = 0.035, *p* < 0.001), and BMR (B = 4.06E-04, *p* < 0.001) all had significant positive effects on ln(BMI) in the combined sample. However, PA (B = 5.25E-06, *p* = 0.078) did not show a significant direct effect.

**Table 4:** Multiple linear regression, gender moderating relation of body composition, PA, BMR with BMI, after adjusting for faculties

Variable	B	SE	Waldc^2^	P	95% CI	
					Lower	Upper
Gender	-0.216	0.041	27.449	< .001	-0.296	-0.135
BFP	0.019	0.001	235.771	< .001	0.016	0.021
Gender ×BFP	-0.002	0.002	1.348	0.246	-0.005	0.001
Gender	0.030	0.111	0.074	0.785	-0.188	0.248
BM	0.190	0.028	47.004	< .001	0.136	0.244
Gender ×BM	0.012	0.043	0.077	0.782	-0.072	0.096
Gender	0.220	0.118	3.475	0.062	-0.011	0.452
MM	0.012	0.001	73.053	< .001	0.010	0.015
Gender ×MM	-0.002	0.003	0.763	0.382	-0.008	0.003
Gender	-0.037	0.016	5.572	0.018	-0.069	-0.006
VFL	0.035	0.002	439.475	< .001	0.032	0.038
Gender ×VFL	0.014	0.003	15.847	< .001	0.007	0.021
Gender	-0.070	0.025	7.894	0.005	-0.119	-0.021
PA	5.25E-06	2.98E-06	3.110	0.078	-5.844E-07	1.11E-05
Gender ×PA	-1.20E-08	6.19E-06	3.759E-06	0.998	-1.215E-05	1.21E-05
Gender	-0.176	0.110	2.558	0.110	-0.392	0.040
BMR	4.06E-04	3.76E-05	116.585	< .001	3.32E-04	4.80E-04
Gender ×BMR	2.29E-04	8.23E-05	7.728	0.005	6.75E-05	3.90E-04

Note. BMI, body mass index; dependent variable: natural logarithm of BMI (InBMI); *BFP *body fat percentage, *BM *bone mass, *MM *muscle mass, *VFL *visceral fat level, *PA *physical activity, *BMR *basal metabolic rate, *SE *standard error, *95% CI* 95% confidence interval

E indicates scientific notation (e.g., 2.287E-04 = 0.0002287 = 2.287 × 10⁻⁴)

*p < 0.05, **p < 0.01, ***p < 0.001

### Gender moderation effects

Interaction terms were introduced (gender × variable) to test for moderation. Most interactions were non-significant: gender × BFP (*p* = 0.246), gender × BM (*p* = 0.782), gender × MM (*p* = 0.382), and gender × PA (*p* = 0.998). However, gender significantly moderated two relationships: (1) VFL and ln(BMI) (B = 0.014, *p* < 0.001), indicates that when the female students increase 1 unit of VFL, their BMI (in log scale) increase 0.014 kg/m^2^ more than male students; (2) BMR and ln(BMI) (B = 2.29E-04, *p* = 0.005), indicates that female students increase 1 unit of BMR, their BMI (in log scale) increase 2.29E-04 kg/m^2^ more than male students. .

#### Graphical interpretation of moderation

**Fig. 2:**
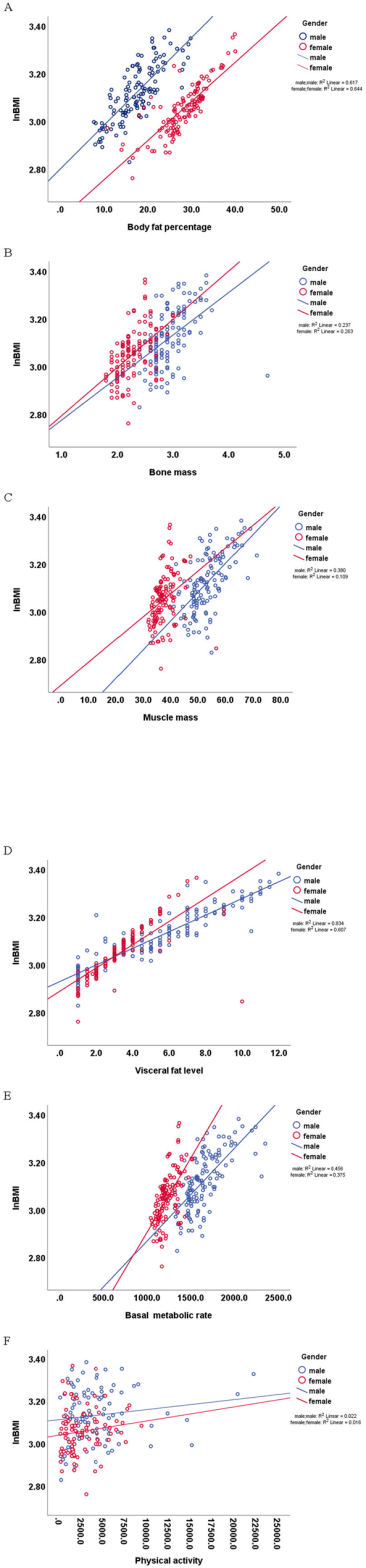
Scatter plot: moderating effect of gender on relation between body composition, PA, BMR, and InBMI Note: blue dot/line = males; red dot/line = females

Graphical analysis further clarified these interactions (Fig. 2): (1)VFL: While VFL was positively correlated with BMI for both gender, an increase in VFL above approximately 3.5 levels was associated with a greater increase in BMI for women than for men. Conversely, maintaining a normal VFL had a more pronounced BMI-lowering effect in women than in men. BMR was also positively associated with BMI for both gender. However, the graph suggests that an increase in BMR may have a more significant impact on the BMI of women compared to men. Additional graphical observations (ignoring other factors) showed that at the same BFP or PA levels, men had higher BMI than women, whereas at the same BM or MM levels, women had higher BMI than men.

## Discussion

The study reveals a concerning prevalence of overweight and obesity (over 25% of participants). A significant sex-based disparity was observed in BMI distributions, with the combined prevalence of overweight and obesity being notably higher in men (36.0%) than in women (15.2%). Specifically, men exhibited twice the rate of overweight (24.0% vs. 12.0%) and nearly four times the rate of obesity (12.0% vs. 3.2%) compared to their female counterparts. This striking difference underscores the critical need for gender-specific approaches in both obesity research and prevention strategies. These results indicate that FM components, specifically BFP and VFL, alongside BMR, play a stronger and more direct role in predicting BMI within this young adult cohort than do FFM components (MM and BM) and PA. Furthermore, the study demonstrated that gender functions as a critical statistical moderator, significantly influencing the effects of VFL and BMR on BMI. In contrast, the predictive effects of BFP, MM, BM, and PA on BMI were found to be invariant across gender.

### Determinants of BMI: the roles of gender, FM, and BMR

Our regression models revealed that gender, FM components (BFP and VFL), and BMR were significantly associated with BMI; MM, BM, and PA did not contribute independently in the final model.

#### Gender as a primary predictor

First, gender emerged as a significant independent predictor of BMI, consistent with established physiological differences and prior findings [11, 39]. This aligns with studies showing that gender more strongly influences the BFP–BMI relationship than age [39], and research noting that BMI changes affect body composition metrics differentially between gender [11]. These observations emphasize that gender-specific reference ranges and clinical interpretations are essential when using BMI to assess body composition and obesity risk.

#### The dominant role of FM components

Second, the substantial main effect of FM on BMI, represented by BFP and VFL, was clearly demonstrated. Since obesity is fundamentally defined by excessive body fat accumulation, the strong influence of BFP on BMI is consistently reported [8, 9, 12]. This reinforces the notion that BMI serves primarily as a surrogate measure of FM accumulation rather than FFM. Reflecting established sexual dimorphism in fat storage [15, 40], our study captured anatomical differences shaped by puberty [14]: (1) VFL showed a stronger correlation with BMI in men, who displayed a higher average VFL index (5.55). (2) BFP showed a stronger correlation with BMI in women, who displayed a higher average BFP (20.08%). These disparities reflect that men are more prone to central/visceral (android) fat accumulation, while women typically favor peripheral/subcutaneous (gynoid) fat storage. Consequently, young men should prioritize monitoring their risk for abdominal/visceral obesity, while young women need to monitor BFP, a metric that may not always be accurately reflected by BMI alone.

#### BMR as a significant predictor

Third, the BMR was significantly associated with BMI. This relationship is physiologically grounded, given that increased BW typically results in greater MM, which consequently elevates BMR [28]. Consistent with this underlying mechanism, prior research has established a positive correlation between resting metabolic rate and several key body composition components, including MM, VFL, and BMI [41].

#### Integrated assessment and gender specificity

In summary, our results support the continued utility of BMI when complemented by precise body composition indicators such as BFP and VFL, along with BMR, for obesity assessment and prevention. Crucially, these findings underscore the necessity of gender-specific weight management, given distinct fat distribution patterns and the differential influence of metabolic rate between young men and women.

It should be noted that the high correlations observed between BMI and fat-related indicators, particularly VFL (VFL vs. BMI, *r* = 0.869), reflect an inherent physiological and conceptual overlap, as BMI is largely driven by overall and visceral adiposity. Multicollinearity was formally assessed and remained within acceptable limits, with VIF values of 5.904 for gender, 4.832 for BFP, 4.763 for VFL, and 4.818 for BMR. These values indicate moderate but non-critical collinearity. Nevertheless, such interrelationships may attenuate the apparent independent effects of closely related predictors, and the regression results should therefore be interpreted with appropriate caution.

#### Limitations of FFM

Our regression models indicated that BM and MM, components of FFM, were not significant independent predictors of BMI. This highlights a methodological limitation of BMI-based assessments, which obscures the specific roles of bone and muscle. While BM supports skeletal health [16, 42] and MM determines metabolic activity [28, 43], their distinct contributions are inadequately reflected when BMI is the sole outcome. This is further supported by the physiological reality that men generally have more MM and less FM than women at the same BMI, likely contributing to observed gender differences in BMR [43]. These results reinforce the need for gender- and body composition–specific assessments that move beyond BMI limitations by fully accounting for BM and MM.

#### Non-significant role of PA

Paradoxically, men reported significantly higher PA levels than women, yet showed higher rates of overweight and obesity. Moreover, our analyses found no significant association between PA and BMI in either gender. These results diverge from prior research, which identified a non-linear relationship (moderate PA beneficial, excessive PA not) [44], and other findings linking only high-intensity PA to reduced obesity risk [24]. However, they align with studies noting substantial variability in the PA-BMI relationship across different populations and gender [45].

The lack of a significant association between PA and BMI in this study may be partly explained by measurement and sample related factors. First, PA was assessed using the self-reported IPAQ-SF during the past one week, which is susceptible to recall and social desirability bias, potentially leading to misclassification of actual activity levels [44]. Second, PA was expressed as total MET-min/week, which may obscure biologically relevant dimensions of activity, such as type, intensity, frequency, and muscle-loading characteristics, that are more strongly associated with adiposity and metabolic outcomes [24, 45]. Consequently, PA alone may not fully explain variations in BMI within this relatively homogeneous cohort of university students. Importantly, PA may not exert a direct or linear effect on BMI, particularly among university students with similar age and health status [44]. Rather, the effect of PA on BW may be mediated through changes in body composition, metabolic efficiency, or energy intake—factors that were not fully captured in the present analysis [44].

In addition, total PA, expressed as MET-min/week and estimated based on participants’ recall of the previous week, may be subject to recall bias. PA is not the sole determinant of BW; other factors, including the intensity and type of PA, dietary habits, and metabolic or hormonal differences, also play important roles in influencing BW. In fact, BMI is calculated by dividing an individual’s weight in kilograms by the square of their height in meters (kg/m²). These findings suggest that the relationship between PA and BMI is complex and context-dependent and warrants further investigation using more detailed and objective assessments of PA [44, 45].

### Moderating role of gender: the relationship between VFL and BMI

From a biological perspective, although men generally exhibit higher VFL, the moderation analysis presented in Fig. 2D reveals a clear sex-specific divergence in the relationship between VFL and BMI. Specifically, at an equivalent VFL of 8, females exhibited a higher BMI (approximately 26 kg/m²) than males (approximately 24 kg/m²). Conversely, at an equivalent BMI of 26 kg/m², females demonstrated a lower VFL (approximately 7.8) compared with males (approximately 9.5). This pattern is primarily attributable to sex-related physiological differences in fat distribution, with females typically having a higher proportion of subcutaneous fat. This biological sex difference suggests that the association between BMI and VFL is more strongly influenced by sex in women than in men. Consequently, at a given level of VFL, women tend to carry a greater proportion of total body fat, resulting in a higher BMI. These findings provide a mechanistic explanation for the significant moderating effect of sex on the association between VFL and BMI observed in Table 4. Our findings are consistent with prior reports demonstrating that BMI is highly correlated with both visceral adipose tissue (VAT) and subcutaneous fat [46]. In addition, our results align with previous studies indicating that BMI adequately captures VAT-associated cardiometabolic and cardiovascular risk in men, but not in women [47]. Collectively, the results underscore that reliance on BMI alone for health risk assessment in women may be insufficient, given their relatively lower VFL.

### Moderating role of gender: the relationship between BMR and BMI

The observed sex-specific moderation of the association between BMR and BMI may reflect underlying differences in metabolic capacity. The moderation analysis presented in Fig. 2E demonstrates a clear divergence in the BMR–BMI relationship between men and women. Men typically exhibit higher absolute BMR than women, largely due to greater MM, but less BFP [26–28]. This biological sex difference suggests that the association between BMI and BMR is more strongly influenced by sex in women than in men. Specifically, at an equivalent BMR of 1500 kcal/day, females exhibited a higher BMI (approximately 24 kg/m²) than males (approximately 21.5 kg/m²). Conversely, at an equivalent BMI of 24 kg/m², females demonstrated a lower BMR (approximately 1400 kcal/day) compared with males (approximately 1750 kcal/day). This pattern is primarily attributable to sex-related physiological differences in BMR. These findings are consistent with perspectives proposed by previous researchers, suggesting that sex-specific effects may have important clinical implications, as MM—a readily modifiable determinant that contributes substantially to BMR—differs between men and women [43].

### Limitations and future research directions

This study has several limitations. First, the cross-sectional design precludes causal inference regarding the relationships among body composition, PA, BMR, and BMI, allowing only the identification of associations. Second, body composition and BMR were assessed using BIA, which is known to be sensitive to hydration status, body geometry, and prediction algorithms [41]. In particular, BIA-derived estimates of VFL may exist measurement bias and may systematically underestimate true visceral fat accumulation when compared with reference methods such as DXA or CT [40]. This potential measurement bias could attenuate observed associations involving VFL. Third, PA was assessed via self-report using the IPAQ-SF and expressed as total MET-min/week, which is susceptible to recall and social desirability bias and lacks information on activity type, intensity, and context. These limitations may partially explain the non-significant association observed between PA and BMI. Moreover, IPAQ-SF only measure the past seven days. There are bias to infer the PA effects. Fourth, the use of BMI as the sole outcome measure for overweight and obesity does not fully capture the heterogeneity of body composition and fat distribution, particularly visceral adiposity, which may differ substantially at similar BMI levels. Fifth, our regression models primarily focused on specific physiological and metabolic factors, which may not capture the full complexity of BMI determinants. Several potentially influential moderators and confounders—including dietary intake, psychological stress, sleep patterns, smoking status, alcohol consumption, and reproductive factors in women (e.g., contraceptive use and menstrual cycle phase)—were not measured, but may affect body composition and BMR. These lifestyle and behavioral factors are known to significantly influence body composition and BMR; their exclusion may limit the comprehensiveness of our insights and result in residual confounding that could affect the observed relationships. Sixth, the study sample was obtained through convenience sampling and consisted exclusively of students from a single university who volunteered to participate. This may limit the generalizability of the findings to other settings or populations.

Future research should address these limitations to strengthen the evidence base. Recommendations include: (1) conducting longitudinal studies to clarify temporal and causal pathways among body composition, PA, BMR, and BMI; (2) recruiting more diverse samples beyond military cadets to enhance external validity; (3) using more precise and objective assessment methods, such as DXA or air displacement plethysmography for body composition, indirect calorimetry for BMR, and accelerometry for detailed PA measurement; (4) incorporating additional indicators of body composition and fat distribution, such as waist-to-hip ratio or waist circumference, alongside BMI; (5) incorporating multi-dimensional data, including diet, stress levels, sleep quality, and lifestyle factors, to provide a more holistic understanding of obesity risk; and (6) investigating gender-specific effects of fat distribution and metabolic rate to inform personalized interventions and reduce the risk of weight regain.

## Conclusions

This study identified a concerning prevalence of overweight and obesity (over 25% of participants), with a markedly higher combined prevalence in men (36.0%) than in women (15.2%). The study findings suggest BFP, VFL, BMR, and gender are significantly associated BMI. Moreover, this study provides evidence that gender plays a significant moderating role in the associations between VFL and BMI as well as between BMR and BMI among university students. The relationships between VFL and BMR and between BMR and BMI are more strongly influenced by sex in women than in men. These findings indicate that researchers and clinicians should consider gender differences when examining the associations between VFL and BMI as well as between BMR and BMI. These observations highlight the need for clinicians to incorporate additional health risk indicators—such as FM, particularly BFP and VFL, as well as BMR—alongside traditional measures (BW and BMI) in obesity assessment and prevention. Moreover, these findings underscore the importance of developing tailored, gender-specific weight management plans and clinical recommendations to more effectively manage obesity and improve metabolic health in university students.

## Supplementary Information

Supplementary Material 1.

## Data Availability

The datasets generated and/or analysed during the current study are not publicly available in order to protect the privacy of the study participants, but are available from the corresponding author on reasonable request.

## Abbreviations

BIA: Bioelectrical impedance analysis
BM: Bone mass
BMI: Body mass index
BMR: Basal metabolic rate
BW: Body weight
FFM: Fat-free mass
FM: Fat mass
IPAQ-SF: International Physical Activity Questionnaire Short Form
MM: Muscle mass
PA: Physical activity
VAT: Visceral adipose tissue
VF: Visceral fat
VFL: Visceral fat level
