# Supplementary material for: Gender as a moderator of the relationships among body composition, physical activity, basal metabolic rate, and BMI in Taiwanese university students
Source: BMC Public Health. 2026 Mar 7;26:1222. doi: 10.1186/s12889-026-26810-w (PMC13081497; doi:10.1186/s12889-026-26810-w)
Supplement: Supplementary file 1 — Supplementary Material 1. [file 12889_2026_26810_MOESM1_ESM.docx]

**Supplementary Table S1** Mean-centered multiple regression analysis to test for the independent contributions of gender, body composition, PA, BMR to InBMI, after adjusting for faculties

| Variable | *B* | SE | β | t | *P* | Collinearity | | Model fit indices | | | |
| --- | --- | --- | --- | --- | --- | --- | --- | --- | --- | --- | --- |
|  |  |  |  |  |  | Tolerance | VIF | *R²* | *adjusted R²* | *F* | *P* |
| **Model 1** | | | | | | | | 0.852 | 0.846 | 135.898 | < .001 |
| **Gender** | -0.031 | 0.018 | -0.126 | -1.732 | 0.085 | 0.147 | 6.807 |  |  |  |  |
| **Body composition** |  |  |  |  |  |  |  |  |  |  |  |
| BFP (%) | 0.008 | 0.001 | 0.454 | 6.641 | < .001 | 0.168 | 5.961 |  |  |  |  |
| BM (kg) | 0.029 | 0.018 | 0.112 | 1.587 | 0.114 | 0.156 | 6.398 |  |  |  |  |
| MM (kg) | 0.001 | 0.002 | 0.083 | 0.482 | 0.631 | 0.026 | 38.191 |  |  |  |  |
| VFL (level) | 0.023 | 0.003 | 0.529 | 8.026 | < .001 | 0.180 | 5.544 |  |  |  |  |
| **PA** (MET-min/week) | 1.82E-06 | 1.33E-06 | 0.048 | 1.609 | 0.109 | 0.896 | 1.116 |  |  |  |  |
| **BMR (kcal)** | 5.69E-05 | 6.44E−05 | 0.130 | 0.882 | 0.379 | 0.036 | 27.512 |  |  |  |  |
| **Model 2** | | | | | |  |  | 0.844 | 0.842 | 350.769 | < .001 |
| **Body composition** |  |  |  |  |  |  |  |  |  |  |  |
| BFP (%) | 0.006 | 0.001 | 0.346 | 8.283 | < .001 | 0.459 | 2.180 |  |  |  |  |
| VFL (level) | 0.025 | 0.002 | 0.585 | 10.444 | < .001 | 0.255 | 3.915 |  |  |  |  |
| **BMR (kcal)** | 1.50E−04 | 2.63E−05 | 0.343 | 5.726 | < .001 | 0.224 | 4.467 |  |  |  |  |

Note: Dependent variable: Mean-center and natural logarithm of BMI (c_InBMI); All predictors (BFP, body fat percentage; BM, bone mass; MM, muscle mass; VFL, visceral fat level; PA, physical activity; BMR, basal metabolic rate) were mean-centered prior to analysis; SE, standard error; 95% CI, 95% confidence interval.

**p* < 0.05, ***p* < 0.01, ****p* < 0.001.
